# Unveiling the potential role of natriuretic peptide receptor a isoforms in fine-tuning the cGMP production and tissue-specific function

**DOI:** 10.1038/s41598-023-47710-8

**Published:** 2023-11-22

**Authors:** Wei Fong Ang, Dan Liao, Cho Yeow Koh, R. Manjunatha Kini

**Affiliations:** 1 Department of Biological Sciences, Faculty of Science, National University of Singapore, Singapore, 117558 Singapore; 2 NUS Graduate School of Integrative Sciences and Engineering, National University of Singapore, Singapore, 119077 Singapore; 3 Department of Medicine, Yong Loo Lin School of Medicine, National University of Singapore, Singapore, 117559 Singapore; 4 Department of Pharmacology, Yong Loo Lin School of Medicine, National University of Singapore, Singapore, 117600 Singapore

**Keywords:** Peptides, Cell signalling, Hypertension

## Abstract

Atrial natriuretic peptide (ANP) is a peptide hormone that regulates blood pressure and volume. ANP interacts with natriuretic peptide receptor-A (NPR-A) to lower the blood pressure through vasodilation, diuresis and natriuresis. Previously, we designed two human ANP analogues, one with exclusively diuretic function (DGD-ANP) and the other with exclusively vasodilatory function (DRD-ANP). Although both ANP analogues interact with NPR-A, their ability to produce cGMP was different. Three alternatively spliced isoforms of NPR-A were previously identified in rodents. Here, we evaluated the putative human isoforms for their cGMP production independently and in combination with WT NPR-A in various percentages. All three NPR-A isoforms failed to produce cGMP in the presence of ANP, DGD-ANP, or DRD-ANP. Co-expression of isoforms with WT NPR-A were found to significantly impair cGMP production. Considering the differential tissue expression levels of all three spliced isoforms in rodents have previously been demonstrated, the existence of these non-functional receptor isoforms may act as negative regulator for ANP/NPR-A activation and fine-tune cGMP production by WT NPR-A to different degree in different tissues. Thus, NPR-A isoforms potentially contribute to tissue-specific functions of ANP.

## Introduction

Endogenous natriuretic peptides (NPs), namely ANP, BNP and CNP, are potent vasoactive peptide hormones that play a crucial role in maintaining the systemic blood pressure, fluid and electrolyte balance^1^. They interact with three NP receptors (NPRs), namely NPR-A, NPR-B and NPR-C. Lack of NPs or their receptors have been associated with hypertension and heart failure^2^. ANP and BNP exert their biological effects via the receptor NPR-A with ANP having a stronger binding affinity than BNP^3,4^. Both NPR-A and NPR-B possess intrinsic guanylyl cyclase activity while NPR-C, which lacks the guanylyl cyclase activity, has been speculated to be a clearance receptor^3,5^.

Previously, we studied KNP isolated from the venom of red-headed krait and showed that K-Ring (conserved NP ring of KNP) exhibits exclusively vasodilatory but not diuretic function^6,7^. We evaluated the roles of three distinct amino acid residues found within the K-ring and the C-terminal and identified the residues that are responsible for these functions^8^. By transferring key amino acid residues to human ANP, we designed two ANP analogues—one exclusively vasodilatory (DRD-ANP) and one exclusively diuretic (DGD-ANP). The separation of diuretic and hemodynamic bioactivity in DRD-ANP and DGD-ANP were demonstrated in anesthetized rats^6,8^ and conscious normal and heart failure models of sheep^9^, showing their potential in precision therapeutic strategies for the treatment of acute decompensated heart failure (ADHF) patients. Both ANP analogues interact with NPR-A and produce cGMP^6^. The diuretic analogue (DGD-ANP) and ANP produce cGMP much faster than the vasodilatory analogue (DRD-ANP). However, the underlying mechanisms behind the distinct, orthogonal pharmacological activity of these ANP analogues remain unclear.

**Figure 1:**
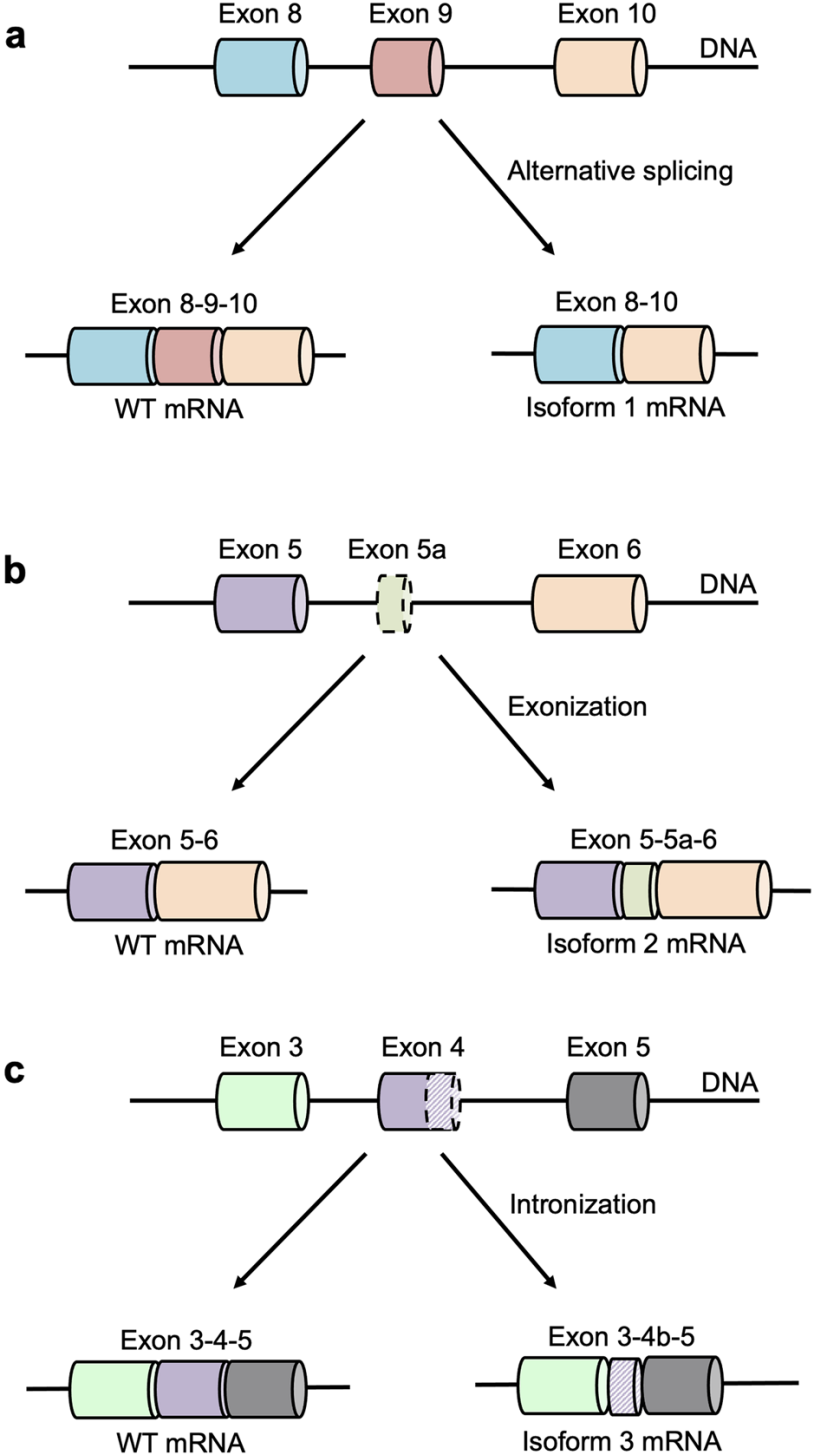
Schematic diagram of all three NPR-A receptor isoforms. **(a)** Alternative splicing of Isoform 1. **(b)** Exonisation of isoform 2. **(c)** Intronization of isoform 3. Top: Introns are represented by the horizonal line. Exons are represented by the colored bars. Bottom: mRNA containing the spliced product for WT NPR-A and the isoform.

In human NPR-A gene, the extracellular domain (450 residues) is encoded by exons 1–6, the transmembrane domain (21 residues) by exon 7, and the intracellular domain (566 residues) by exons 8–22. The intracellular domain contains the protein kinase-like domain (exons 8–15), and the guanylyl cyclase domain (exons 16–22)^10^. NPR-A exists as a homodimer in the absence of its ligand and binding of its ligand does not cause further aggregation of the receptors^2,11^. Ohyama et al*.* reported the first alternative forms of NPR-B in rat brain^12^. Subsequent evaluation showed a similar isoform of NPR-A with a similar 75 bp deletion in the exon 9 (Fig. 1a). This NPR-A isoform was less than 1% of total NPR-A transcripts in various tissues including 0.7% in skeletal muscles, and 0.6% in the testis^13^. The deleted region encodes for the ATP binding site within the kinase-like homology domain. This segment contains four phosphorylation sites^14^ and plays a role in repressing the constitutive activity of particulate guanylyl cyclase^15^. The guanylyl cyclase activity of NPR-A probably remains repressed due to the deletion as the ATP binding fails to initiate the conformational change in the protein kinase-like domain leading to the guanylyl cyclase inactivation through the disruption of interaction between the two intracellular domains^16,17^.

In 1995, a second NPR-A isoform containing a tripeptide (Pro-Cys-Gln) insertion in the extracellular juxtamembrane was identified^18^. This isoform is expressed at ~ 1–2.5% of NPR-A transcripts in rat renal papilla and adrenal, two primary target organs of ANP. The tripeptide insertion is due to splicing of a ‘new’ exon (exon 5a) located within the fifth intron between exons 5 and 6 (Fig. 1b). This process is known as exonization^19^. The impact of this insertion on ligand binding or cGMP production has not been evaluated.

A third NPR-A isoform containing a differentially spliced exon 4 resulting in a 51-bp deletion, which would encode for amino acid residues at the membrane-distal region of the extracellular domain was identified (Fig. 1c)^20^. The formation of this isoform is a process known as intronization where an exon is retain as an intron^19^. This isoform is expressed at less than 10% of the full-length NPR-A transcript in all the tested murine tissues including brain, spleen and testes. This shorter version of NPR-A was found to be a non-functional guanylyl cyclase receptor^20^. The deletion of the 51 bp (17 residues) is far away from the ligand-binding site, and this region encodes for a major part of the $\alpha$-helix 10 in the membrane distal region that is involved in the receptor dimerization^20^. This alternatively spliced isoform is able to form a heterodimer with the full-length NPR-A but in a different conformation, probably resulting in the loss of guanylyl cyclase function. Expression of both the spliced isoforms with the full-length NPR-A results in a decrease in the cGMP production, suggesting the spliced isoforms may serve as a negative regulator for NPR-A activation^20^. The presence of these three alternately spliced isoforms of NPR-A^13,18,20^ and their tissue-specific expression adds to the complexity of NP biology. To keep it simple, we labelled them as isoform 1, 2 and 3, respectively, in this paper (Fig. 1).

The three NPR-A receptor isoforms identified in rodents and their differential tissue expression suggest their involvement in regulating the function of full-length wild-type (WT) NPR-A. Similar splicing isoforms could be found in human. Here, we evaluate the effects of ANP and its two analogues (DGD-ANP and DRD-ANP) on WT NPR-A and NPR-A isoforms on cGMP production. Our results show that these isoforms fail to produce cGMP in the presence of either the ANP or its two ANP analogues. The presence of even a small fraction of these inactive isoforms together with WT NPR-A disproportionately reduced overall production of cGMP in cell-based assays. Therefore, if indeed expressed, they may play a role of fine-tuning the functions of ANP and its ANP analogues depending on their expression levels in various tissues.

## Results

### Isoforms 1 and 3, but not isoform 2, may be found in humans and sheep

**Figure 2:**
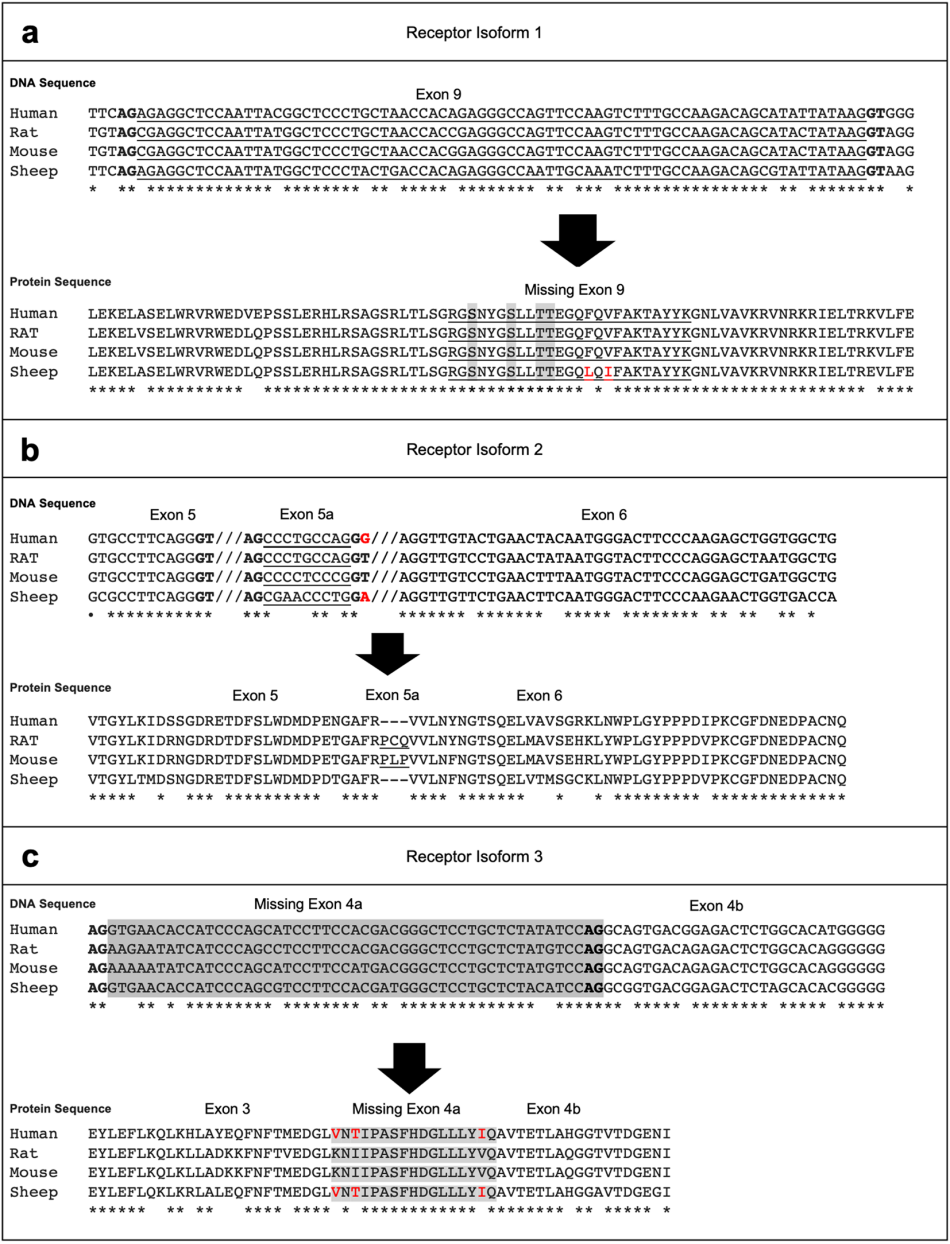
Alignment of NPR-A nucleotide and amino acid sequences of human, rat, mouse and sheep. **(a)** NPR-A receptor isoform 1. The deleted exon 9 is underlined and the residues involved in phosphorylation are highlighted in grey. Differences in amino acid residues are indicated by red font. **(b)** NPR-A receptor isoform 2. Additional insertion of 9 nucleotides and three amino acid residues as exon 5a are underlined. Differences in the nucleotides are indicated by red bold font. The absence of critical GT sequence at 5’ end in human and sheep genes indicates that isoform 2 may not exist in them. **(c)** NPR-A receptor isoform 3. The deleted portion of exon 4 is highlighted in grey. Nucleotides AG in bold represent the sequence recognised by spliceosomes. Differences in the amino acid residues within the missing segments are indicated by red font.

All three isoforms of NPR-A were identified in rodents^13,18,20^. The DNA and amino acid sequences of these isoforms in rat, mouse, human and sheep were aligned for examination. The corresponding deleted exon 9 (75 bp) sequence in isoform 1 is conserved across these species (Fig. 2a). The presence of universal “AG” and “GT” splice sites shows the potential existence of this isoform as a result of an alternative splicing event. Four potential phosphorylation sites (Ser506, Ser510, Thr513 and Thr514)^21^ identified within this deleted exon 9 will be lost in isoform 1 (Fig. 2a).

For isoform 2, which is detected previously in rat, the corresponding new exon 5a (9 bp) sequence complete with “AG” and “GT” splice sites can be identified within the fifth intron of the mouse gene (Fig. 1b). Thus, NPR-A isoform 2 may also occur in mouse. Interestingly, the new exon 5a in mice encodes a different tripeptide Pro-Lys-Pro, unlike the Pro-Cys-Gln in rat. In contrast, no “GT” sequence is located downstream of the predicted exon 5a in human and sheep NPR-A genes (Fig. 1b). Thus, spliceosome cannot recognize the 3’ end of exon 5a and NPR-A isoform 2 will not be expressed in human and sheep unless a single mutation creates the “GT” at the 3’ end of exon 5a.

NPR-A receptor isoform 3 shows the presence of two “AG” splice sites at both upstream and downstream of exon 4a (Fig. 2c). This allows the spliceosome to recognize the upstream “AG” or the downstream “AG” of exon 4a to create either the full-length WT NPR-A or the truncated mRNA (51 bp shorter isoform 3), respectively. The sequence of exon 4a is fully conserved in both rat and mouse but differ at three residues in human and sheep protein (Val314, Thr316 and Ile329). Thus, the alignment of nucleotide and amino acid sequences showed that only isoform 1 and 3 can be expressed in human and sheep by alternative splicing whereas all three isoforms can be expressed in the rodents.

### Effects of ANP on cGMP production by receptor isoforms

**Figure 3:**
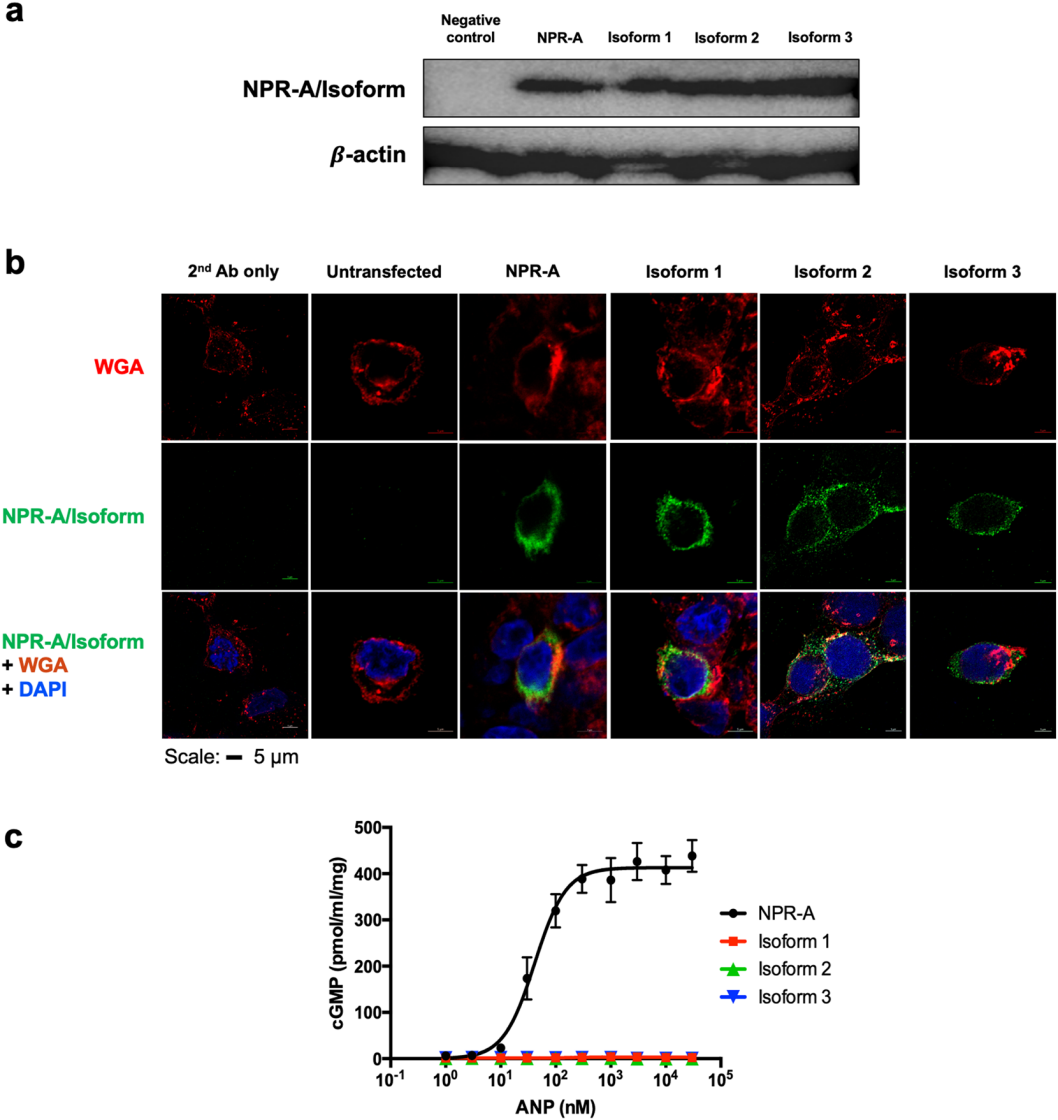
NPR-A and receptor isoforms are expressed on the surface of the transfected cells in similar quantities and only cells containing WT NPR-A produce cGMP. **(a)** Western blot studies. Cells transfected with empty pCMV-4 plasmid were used as the negative control. $\upbeta$-actin was used as the loading control. Full-size blot can be found in Supplementary Fig. S4a. **(b)** Immunofluorescence studies. The nuclei were stained blue with DAPI. Top panel: Cell surface glycoproteins were stained with WGA as the cell membrane marker. Middle panel: staining with antibodies targeting NPR-A/Isoforms. Bottom panel: Merge images show the co-localization of the receptors with the cell membrane marker. Untransfected cells and cells stained with only the secondary antibody were used as the two negative controls. Scale: 5 $\mu$ m; N = 6. Also see Supplementary Videos 1 to 6 for the 3D z-stack. (C) Effects of ANP on cGMP production by receptor isoforms. The cGMP production induced by various concentrations of ANP was determined by ELISA. Each point represents average ± S.D. of quadruplicates. These experiments were repeated on three independent days.

The three isoforms were created from the WT NPR-A plasmid using site-directed mutagenesis and the plasmid containing either the WT NPR-A or the respective isoforms were transfected separately into DLD-1 cells. 800 ng of the respective plasmids was used to achieve 90% transfection efficiency (Supplementary Fig. S1). We performed western blot studies to semi-quantitatively evaluate the expression of WT and all three NPR-A isoforms using the house keeping protein, $\upbeta$-actin as the control. The presence of similar size protein bands suggests that DLD-1 cells show similar expression level of WT NPR-A and its isoforms (Fig. 3a, Supplementary Fig. S2a). To exhibit their function, receptors must be expressed on the cell surface. Therefore, we used immunofluorescence method to evaluate the expression of WT and all three NPR-A isoforms on the cell surface. We performed co-localization experiments using (a) the primary antibody that recognizes extracellular segment that is common among NPR-A and its isoforms, and (b) WGA conjugate that recognizes cell surface glycoproteins as the cell membrane marker. The results from these immunofluorescence studies showed co-localisation of the respective receptors and cell membrane marker, indicating that the WT and all receptor isoforms were expressed on the surface of the cells (Fig. 3b, Supplementary Fig. S2b,c).

To test the function of WT and all NPR-A isoforms, we treated DLD-1 cells transfected with respective receptors with various doses of ANP and measured cGMP produced. All peptides were synthesized by solid phase peptide synthesis (Supplementary Fig. S3). The cells transfected with WT NPR-A showed dose-dependent increase in cGMP production (Fig. 3c). cGMP production showed an EC_50_ value of 20.9 nM and reached a plateau at ~ 300 nM ANP. However, no cGMP production was observed in the cells transfected with any of the three isoforms. This suggests that all three NPR-A receptor isoforms were not able to produce cGMP, unlike the WT NPR-A. Earlier published data also showed the inability of isoform 3 to produce cGMP^20^.

### Role of receptor isoforms on ANP-induced cGMP production by WT NPR-A

**Figure 4:**
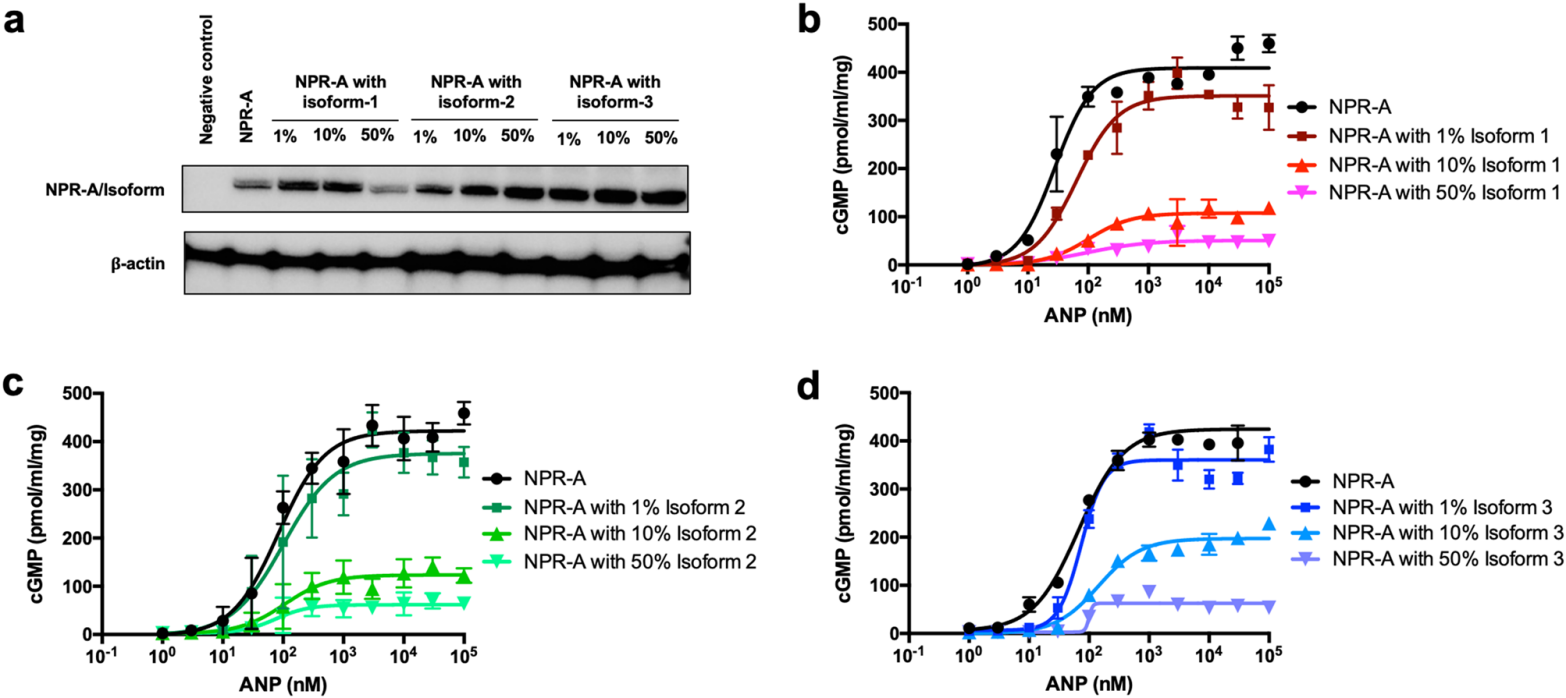
Impact of co-transfection of receptor isoforms with WT NPR-A in 1%, 10% and 50% on cGMP production by WT NPR-A. The cGMP production induced by various concentrations of ANP was determined by ELISA. **(a)** Western blot illustrating the expression of both the receptors and the WT NPR-A in various percentage of co-transfection. Full-size blot can be found in Supplementary Fig. S4b. **(b)** Co-transfection of receptor isoforms 1 with WT NPR-A. **(c)** Co-transfection of receptor isoform 2 with WT NPR-A. **(d)** Co-transfection of receptor isoform 3 with WT NPR-A. Each point represents average ± S.D. of triplicates. These experiments were repeated on three independent days.

To determine the effect of the spliced isoforms on the WT NPR-A, plasmid containing each of the receptor isoforms are co-transfected with the WT NPR-A into the cells at different ratios (1%, 10% and 50%). Western blotting showed some variations in receptor expression level under different co-transfection conditions, indicating that the expression of WT NPR-A plus isoforms on cell surface may not be always consistent (Fig. 4a, Supplementary Fig. S2d). However, we think the data are reasonably robust since the cGMP assay were performed at equilibrium at a single time point (30 min post stimulation), and the level of cGMP production appeared to start to saturate at similar concentrations of ANP (~ 10 nM)^3^ under all different co-transfection conditions. All three receptor isoforms displayed a fall in cGMP production in a dose-dependent manner (Fig. 4b–d). The higher the concentration of receptor isoforms, the lower the cGMP produced. Ratio used in the co-transfection experiment reflects the abundance of each of these three receptor isoforms reported in the rodents. At 1% co-transfection with isoform 1, the cGMP production of WT NPR-A falls by approximately 10.6% and this decreases further to 70.6% when the concentration of the receptor isoform increases to 10% (Fig. 4b). At 50% co-transfection with isoform 1, the cGMP production of NPR-A reduced by 87.4%. cGMP production was decreased by 8.0% at 1% co-transfection, 69.8% at 10% co-transfection and 84.1% at 50% co-transfection with isoform 2 (Fig. 4c). Lastly, 1% co-transfection with isoform 3 lowered the cGMP production by 22.6% (Fig. 4d). Co-transfection with 10% isoform 3 reduced the cGMP production by slightly more than half (54.3%). When 50% of isoform 3 was co-transfected, the cGMP production fell by 86.2%. It also appears that small percentage of all three receptor isoforms have a rather large impact on the activity of the WT NPR-A.

### Effects of ANP analogues on cGMP production by receptor isoforms

**Figure 5:**
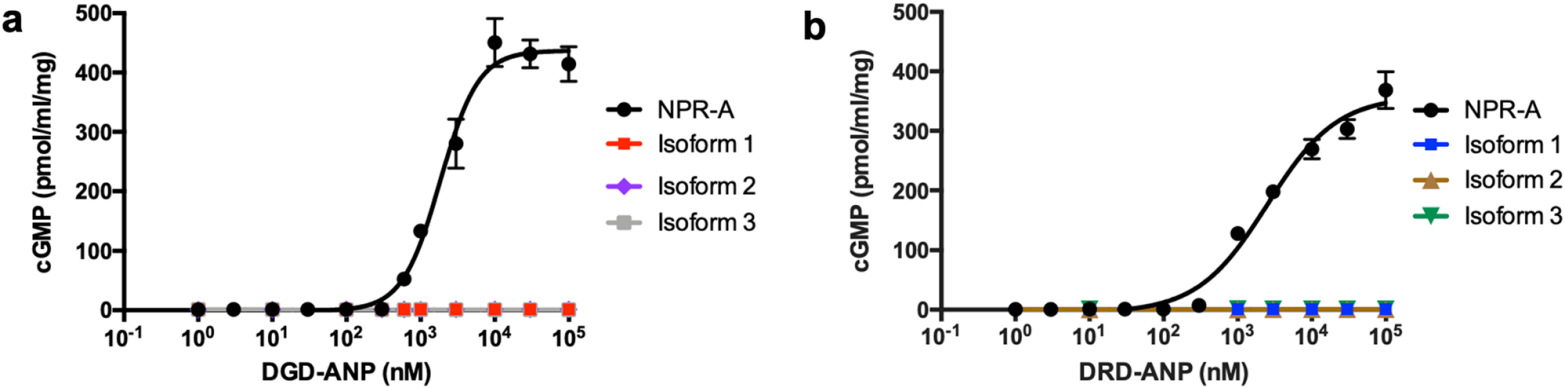
NPR-A receptor isoforms are non-functional guanylyl cyclases in the presence of **(a)** DGD-ANP and **(b)** DRD-ANP. The cGMP production induced by various concentrations of ANP was determined by ELISA. Each point represents average ± S.D. of triplicates.

Cells transfected with WT NPR-A or any of the three receptor isoforms were incubated with the two ANP analogues (DGD-ANP and DRD-ANP) and measured the cGMP produced. Only WT NPR-A transfected cells produced cGMP in a dose-dependent manner when incubated with either DGD-ANP or DRD-ANP (Fig. 5). No cGMP was detected in cells that were transfected with the three receptor isoforms. These results show that all three receptor isoforms failed to produce cGMP in the presence of both ANP analogues, similar to ANP.

### Role of receptor isoforms on DGD-ANP- or DRD-ANP-induced cGMP production by WT NPR-A

**Figure 6:**
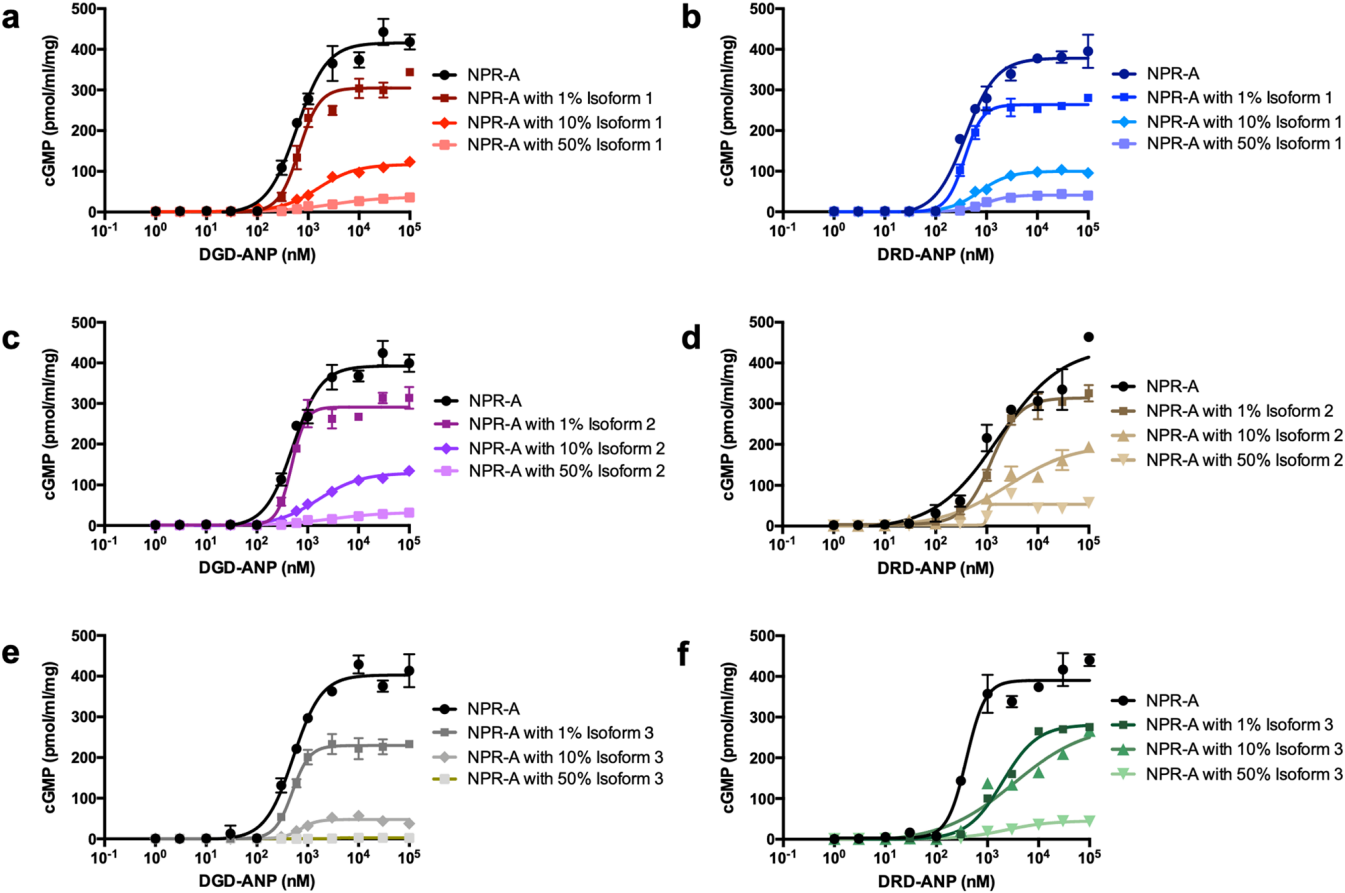
Receptor isoforms reduce cGMP production of WT NPR-A in the presence of ANP analogues. The cGMP production induced by various concentrations of ANP was determined by ELISA. **(a)** & **(b)** Co-transfection of receptor isoforms 1 with WT NPR-A in the presence DGD-ANP and DRD-ANP respectively. **(c)** & **(d)** Co-transfection of receptor isoform 2 with WT NPR-A in the presence DGD-ANP and DRD-ANP respectively. **(e)** & **(f)** Co-transfection of receptor isoform 3 with WT NPR-A in the presence DGD-ANP and DRD-ANP respectively. Each point represents average ± S.D. of triplicates.

We evaluated cGMP production induced by DGD-ANP and DRD-ANP in DLD-1 cells co-transfected with WT NPR-A and various percentages of all three isoforms. As with ANP, DGD-ANP and DRD-ANP produced a similar trend of decreased cGMP production by WT NPR-A in the presence of each of the NPR-A isoforms (Fig. 6). At 1% co-transfection with isoform 1, cGMP production decreased to 27.8% for DGD-ANP and 31.5% for DRD-ANP. This decreased further to 76.9% (DGD-ANP) and 72.8% (DRD-ANP) at 10% co-transfection, and 91.4% (DGD-ANP) and 88.5% (DRD-ANP) at 50% co-transfection (Fig. 6a,b). Similarly, co-transfection with 1% isoform 2 lowered the cGMP production of WT NPR-A by 24.8% (DGD-ANP) and 20% (DRD-ANP) respectively (Fig. 6c,d). At 10% co-transfection, the WT NPR-A activities decreased by 66.2% (DGD-ANP) and 51.8% (DRD-ANP). At 50% co-transfection, the WT NPR-A activities decreased by 92.1% (DGD-ANP) and 85.8% (DRD-ANP) (Fig. 6c,d). Co-transfection with 1% isoform 3 decreased the cGMP production of WT NPR-A by 41.8% (DGD-ANP) and 27.4% (DRD-ANP) (Fig. 6e,f). This further decreased to 87% (DGD-ANP) and 30% (DRD-ANP) at 10% co-transfection. At 50% co-transfection, WT NPR-A activities decreased by 99.3% (DGD-ANP) and 88.6% (DRD-ANP) (Fig. 6e,f).

In the presence of either of the ANP analogues, our results show that 1% co-transfection of isoform 1 and 2 leads to reduction in cGMP production about 2–3 times more than that ANP (20–31.5% for ANP analogues compared to 8–10.6% for ANP). In contrast, in the presence of 10% co-transfection of isoform 3, cGMP production induced by DGD-ANP-WT-NPR-A interaction (87% reduction) was greatly reduced compared to ANP-WT-NPR-A interaction (54.3% reduction) which is greater than DRD-ANP-WT-NPR-A (30% reduction). Thus, suggesting that while both ANP analogues are more potent than ANP in reducing cGMP production when isoform 1 or 2 are present, they differ in their abilities to reduce cGMP production in the presence of isoform 3 with DGD-ANP causing a greater reduction than ANP and DRD-ANP having a weaker reduction than ANP.

## Discussion

ANP and its ANP analogues differentially regulate systemic blood pressure, fluid and electrolyte balance through their interactions with NPR-A^8^. As the production of secondary messenger cGMP controls the physiological response, here we evaluated the contribution of the three NPR-A receptor isoforms previously documented^13,18,20^. These three isoforms exist in rodents by alternative splicing mechanisms. We analyzed human and sheep NPR-A gene sequences to show that isoforms 1 and 3 can theoretically be produced by alternate splicing. However, isoform 2 cannot formed from human and sheep genes unless a single key mutation occurs at the end of the intron 5. Isoform 1 is formed by alternative splicing with the removal of the entire exon 9. The deleted portion consists of four potential phosphorylation sites and three out of these four amino acid residues (Ser506, Ser510 and Thr513) have been shown to regulate the activation of NPR-A^14,21^. However, the importance of phosphorylation of this domain remains controversial. On one hand, transfecting cells with NPR-A lacking the entire kinase-like homology domain results in a constitutively active form of the receptor^16^. On the other hand, it was found that mutating any of the seven phosphorylation sites (Ser497, Ser498, Thr500, Ser502, Ser506, Ser510 and Thr513) to Ala in mammalian cell lines causes a decrease in both phosphorylation and ANP-dependent cGMP production^21^. This study showed that deletion of the four phosphorylation sites (Ser506, Ser510, Thr513 and Thr514) results in a non-functional receptor (Figs. 2a and 4). In addition, when considering the abundance of the receptor isoforms reported in the rats (less than 1% of the total NPR-A transcripts), the cGMP assay showed that presence of just 1% isoform 1 reduced the WT NPR-A activity by 10.6% (Fig. 5b). Even low level expression of isoform 1, if found in human tissues, could greatly affect the function of WT NPR-A in the system.

Isoform 2 is created by exonization with the addition of a new exon 5a and this encodes for three amino acid residues, PCQ and PLP in rat and mouse respectively (Fig. 2). Proline residues induce kinks in the protein structure and have been shown to be involved in influencing the conformation of short loops containing 2 to 10 residues in polypeptide chains^22^ and critically affects protein–protein interaction and function^23^. The positioning of Pro in the signal sequence is known to alter the secretion of a protein^24^. Hence, introducing an additional Pro into the polypeptide may possibly alter the structure of the NPR-A extracellular domain and thus, possibly affects its function. Presence of Cys in rat may interact with existing disulphide linkages and potentially alter the structure and function of the receptor. Our study demonstrates that cells containing only isoform 2 do not produce cGMP upon addition of ANP or its ANP analogues (Fig. 4). Further, this isoform also reduces the cGMP production by WT NPR-A (Fig. 5c). Considering the percentage of isoform 2 detected in the rats (1–2.5% of the total NPR-A transcript), our data showed that 1% co-transfection gave 8.0% reduction in WT NPR-A activity while 10% co-transfection gave 69.8% reduction in cGMP production (Fig. 5c). Thus, 1–2.5% of receptor isoforms could have a significant reduction in the NPR-A activity ranging from 8 to 69.8% reduction.

Lastly, isoform 3 is created from the partial deletion of exon 4 (intronization). Our data supports the functional study done by Hartmann et al*.* despite the use of different cell line (DLD-1 vs. HEK293) and a higher concentration of ANP used per well (0.8 $\upmu$ g vs. 0.2 $\upmu$ g ANP/well)^20^. Transfection of isoform 3 alone into DLD-1 cells fails to produce cGMP upon addition of ANP or its ANP analogues (Fig. 4). Further, this isoform also reduces the cGMP production by WT NPR-A (Fig. 5d). cGMP assay shows that presence of 10% isoform 3 can reduce the cGMP production by 54.3%. Given that the abundance of isoform 3 in the mice was about 10% of the total NPR-A transcript, having 10% of the endogenous total NPR-A transcript as isoform 3 could have a great reduction in the WT NPR-A activity *in-vivo*.

Interestingly, all three isoforms, the degree of reduction in the cGMP production by WT NPR-A is greater than expected. For example, considering the possibility that these isoforms can form a heterodimer with the WT receptor, theoretically, 1% co-transfection could reduce the activity of WT receptor by 1% or less depending on the number of isoforms that bind with the WT NPR-A to form heterodimer. Hence, 99% out of the 100% WT NPR-A will remain functional with 1% hindered by the isoforms. However, based on the cGMP assay, all three isoforms at 1% co-transfection displayed 8%-22% reduction in the cGMP production. Similar observations were made also for the 10% and 50% co-transfection respectively. The mechanism that leads to much higher reduction cGMP production is unclear. These spliced isoforms are differentially expressed in various tissues (0.5%-10% NPR-A transcripts) in rodents^13,18,20^. All three isoforms were detected in the kidney, compared to other organs. Within the types of cells found in the kidney, a single cell RNA-seq data suggests that 52.6% NPR-A transcripts are found in podocytes, 9.69% in endothelial cells, and 5.46% in collecting duct transitional cells^25^. However, this data does not evaluate the expression levels of these isoforms. Despite the lack of such data, we have observed that the variations in ratios between WT NPR-A and these isoforms may significantly affect the cGMP production locally. Therefore, the function of ANP as an agonist could be highly variable in different tissues depending on the expression level of different isoforms or changes due to disease states. As natriuretic peptides (e.g., anaritide and nesiritide)^26^ are being used in clinics and in the development pipeline^27^, understanding of the regulation of ANP (or other analogues)-NPR-A interactions could provide further insights to the pharmacology of these molecules. Furthermore, we cannot rule out that in the *in-vivo* setting, the regulatory effect of the three receptor isoforms may also be influence by other circulating biological molecules such as angiotensin. Thus, careful evaluations are required to understand the subtle and complex network of regulation of the NPR-A activity in the kidney. Nevertheless, this study illustrates the potential negative regulatory role of the existence of the NPR-A receptor isoforms and how the presence of these isoforms even at a low percentage may be sufficient to cause a significant reduction in the cGMP production and WT NPR-A function.

We also determined the cGMP production induced by two ANP analogues, DGD-ANP and DRD-ANP, which exhibit distinct, orthogonal pharmacological activity^7,8^. As expected, both ANP analogues displayed reduction in WT NPR-A activities when WT NPR-A was co-transfected with any of the three receptor isoforms at various ratios similar to ANP (Fig. 6). However, the extent of reduction was greater as compared to ANP, possibly due to the lower affinity of these ANP analogues to the NPR-A^8^.

The current study is not without limitations. So far, neither the presence of these isoforms in human tissues nor their functional studies has been reported. We analyzed nucleotide sequences of NPR-A genes to show that two-out-of-three rodent isoforms may be expressed in sheep and human. In human cells, transfected plasmids with the coding sequences of all three isoforms can be transcribed and translated into NPR-A receptors and being expressed on cell surface. However, the presence of the mRNAs transcripts of these isoforms in various human tissues and their relative abundances have not been documented. Nonetheless, our analysis of the data from a published study of sheep renal transcriptomes detected the transcripts for isoforms 1 and 3 (data not shown)^28^. Here, we have demonstrated that these isoforms are non-functional and, if present, may cause disproportionate reduction of cGMP production. Direct identification of these and other isoforms in human tissues and quantification of their abundance would be needed to provide further insights. In addition, dimerization of WT NPR-A is an essential step in receptor activation^29^. In the current study, we have not verified if WT NPR-A can form a stable dimer with any one of the isoforms. However, previous studies by Hartmann et al*.* have shown that isoform 3 forms heterodimers with WT NPR-A, exhibiting similar binding affinity^20^. Based on this, we assume that isoforms 1 and 2 may also form heterodimers and retain similar binding affinity towards ANP. Therefore, appropriate cautions should be applied when interpretating the results due to the above limitations.

## Conclusion

In conclusion, if expressed in human, the three NPR-A spliced isoforms would most likely play a negative regulatory role in fine-tuning the cGMP production and hence, alter the function of ANP (or its ANP analogues) and WT NPR-A complexes. This fine-tuning may be one of the mechanisms responsible for subtle differences in the rates of cGMP production and altered pharmacology at various target tissues. This in turns could contribute to tissue specific functions of ANP, highlighting the importance of receptor isoforms in regulating the functions of ANP.

## Materials and methods

### Materials

Anti-natriuretic peptide receptor A/GC-A polyclonal antibody (ab14356; Abcam); DLD-1 human colon cell line (ATCC; CCL-221); Blotting-Grade Blocker (Bio-Rad); Precision Plus Protein Dual Color Standards ladder (Bio-Rad); Trans-Blot Turbo kit (Bio-Rad); Tyr-preloaded Wang resin (Creosalus); RPMI-1640 (Gibco); cGMP Competitive ELISA kit (Invitrogen); ProLong™ Gold Antifade Mountant reagent (Invitrogen); Simply Blue (Lifetech); C-Chip Disposable hemocytometer (NanoEnTek); Q5® Site-Directed Mutagenesis kit (NEB); 3-isobutyl-1-methylxanthine (Sigma); Dioxa-1,8-octane-dithiol (TCI); trifluoroacetic acid (TCI); triisopropylsilane (TCI); Acetonitrile (ThermoFisher Scientific); Bicinchoninic acid assay (ThermoFisher Scientific); Lipofectamine 3000 (ThermoFisher Scientific); *N,N*-diisopropylcarbodiimide (ThermoFisher Scientific); *N,N-*dimethylformamide (ThermoFisher Scientific) were purchased.

### Multiple sequence alignment of isoforms

Sequences of all three receptor isoforms were obtained from the respective papers^13,18,20^. Using the given sequence in the rodents, corresponding sequences from human and sheep were identified through NCBI-BLAST. Multiple sequence alignment was performed using the Clustal Omega Software (https://www.ebi.ac.uk/Tools/msa/clustalo/).

### Cloning of NPR-A and three isoforms

A plasmid containing rat NPR-A in a pCMV-4 backbone, gifted by Dr. Ruey-Bing Yang (Academia Sinica), was used as the NPR-A template. All three receptor isoforms plasmids were created by modifying the NPR-A sequence in the template plasmid using Site-Directed Mutagenesis (SDM) following the protocol from the Q5® Site-Directed Mutagenesis kit. All plasmids were cloned and expanded in NEB 5 $\alpha$ competent cells. DNA sequencing was done to confirm the success of the SDM. Primers used to clone isoform 1 are 5’—tga ccc tga gtg ggg gca acc ttg tgg c—3’ and 5’—gcc aca agg ttg ccc cca ctc agg gtc a—3’. primers used to clone isoform 2 are 5’ –gac ggg tgc ctt cag gcc ctg cca ggt tgt cct gaa cta ta—3’ and 5’ –tat agt tca gga caa cct ggc agg gcc tga agg cac ccg tc—3’. Primers used to clone isoform 3 are 5’—gtg gag gat ggc ctg gca gtg aca gag act—3’ and 5’—agt ctc tgt cac tgc cag gcc atc ctc cac—3’.

### Peptide synthesis

ANP, DGD-ANP and DRD-ANP were synthesized using Fmoc-based solid-phase peptide synthesis and on Tyr-preloaded Wang resin. Synthesis was carried out in the presence of *N,N-*dimethylformamide (DMF) as the general solvent and the peptide was synthesized from the C-terminal to the N-terminal. Amino acids were coupled one at a time to the Tyr-preloaded Wang resin using 0.5 M *N,N*-diisopropylcarbodiimide (DIC) as the activator and 0.1 M *N,N-*diisopropylethylamine (DIEA) in 1 M Oxyma as activator base. After each coupling, the Fmoc group was removed using 10% (v/v) piperazine dissolved in ethanol:*N*-Methyl-2-pyrrolidone at 1:9 ratio. To avoid the formation of aspartimide as side products, Fmoc-Asp(OtBu)-(Dmb)Gly-OH dipeptide was used for DGD-ANP synthesis. After the completion of synthesis, peptides were cleaved from the resin for 3 h at room temperature using a cleavage cocktail containing trifluoroacetic acid (TFA), triisopropylsilane (TIS), water and dioxa-1,8-octane-dithiol (DODT) (94/2.5/2.5/2.5). The peptides were precipitated using cold, dry, diethyl ether and resuspended in 0.1% TFA solution for purification. ANP and its analogues were purified using RP-HPLC on Jupiter column C18 (5 $\upmu$ m, 300 Å, 10 mm × 250 mm) with 0.1% TFA as buffer A and 0.1% TFA with 80% acetonitrile (ACN) as buffer B. The purity of the peptide was assessed using ESI–MS and pure fractions were freeze-dried. The freeze-dried samples were subsequently subjected to air oxidation in 50% ethanol, pH 8 for 3 h at room temperature with constant mixing. The oxidized peptides were purified by RP-HPLC and the mass determined by ESI–MS using an LCQ Fleet™ ion trap mass spectrometer (ThermoFisher Scientific). The fractions containing the homogenous oxidized peptides were freeze-dried and used in the subsequent experiments.

### Transfection of plasmids containing WT NPR-A and isoforms 1, 2, and 3

DLD-1 cells were cultured in complete RPMI-1640 medium supplemented with 10% HI-FBS and 1% Penicillin–Streptomycin (P/S). Cells were sub-cultured every 3 days using trypsinization. DLD-1 cells (0.2 million) were seeded per well in 24-well plates to attain near 70% confluent before transfection was performed following the manufacturer’s protocol. Plasmids of interest was incubated with lipofectamine 3000 in a 1:1 ratio before the mixture was added to the cells. For single transfection, 0.8 $\upmu$ g of the plasmid of interest was used. For co-transfection, the NPR-A WT plasmid was fixed at 0.8 $\upmu$ g and the receptor isoforms were mixed with the WT plasmid according to their given percentages. For 1% co-transfection, 0.8 $\upmu$ g of NPR-A WT plasmid was mixed with 0.00808 $\upmu$ g of receptor isoforms. For 10% co-transfection, 0.8 $\upmu$ g of NPR-A WT plasmid was mixed with 0.0889 $\upmu$ g of receptor isoforms. For 50% co-transfection, 0.8 $\upmu$ g of NPR-A WT plasmid was mixed with 0.8 $\upmu$ g of receptor isoforms. From each of these DNA mixtures with their respective percentages of isoforms, a fixed volume was then mixed with the same volume of lipofectamine. The cells were subsequently allowed to grow for 6 h to allow the cells to take up the plasmids. After 6 h incubation, the transfection mixture was replaced with fresh complete media before the transfected cells were used for downstream experiments.

### GFP transfection efficiency test

Different concentrations of GFP plasmid (8 ng, 80 ng, 400 ng and 800 ng) were transfected separately DLD-1 cells following the transfection protocol. Transfected cells were viewed under the fluorescence microscope and the intensity of the fluorescence was captured as an image. White light was used to capture the image of cells in each well. Cells that had been successfully transfected were identified through the comparison of the fluorescence imaging and the white light imaging.

### Whole cGMP assay

Cells were transfected following the transfection protocol above and incubated for another 16 h overnight. They were subsequently treated with 0.5 mM 3-isobutyl-1-methylxanthine (IBMX) for 30 min followed by different concentrations of ANP peptides for another 30 min. Thereafter, the cells were lysed in 0.1 M HCl with 1% Triton X-100 for 20 min and the lysis of the cells were confirmed under the microscope. The lysed cells were centrifuged, and the cell lysate was collected and assayed using cGMP competitive ELISA kit, following the manufacturer’s protocol. Total protein content of three wells were determined using Bicinchoninic acid assay and used for normalizing across the different 24-well plates. Expression of the receptors in 3 wells were determined using Western blotting.

### Coomassie blue staining and western blotting

Samples were stained using 5 × SDS loading dye (250 mM Tris–HCl, pH 6.8; 10% SDS; 30% glycerol; 0.02% bromophenol blue) and reduced by 0.6 M dithiothreitol (DTT). This was followed by denaturation (linearization) through heating at 95 °C for 10 min. Subsequently, samples were loaded on a 12.5% SDS page gel and run at constant 60 V for 120 min. Upon completion of the SDS gel electrophoresis, gel was subjected to overnight Coomassie blue staining using the Simply Blue stain. Thereafter, the gel was de-stained through repeated washing using tap water until the bands and the ladder were visible. Image of the gel was then captured by a SynGene CB software. For Western blotting, after gel electrophoresis, gel was stacked in a sandwich manner following the Trans-Blot Turbo system manufacture protocol. Proteins were transferred using the semi-dry technique onto a PVDF membrane. This membrane was washed for 30 min using 1 × PBS containing 1% Tween 20 (PBS-T). Blocking of the membrane was carried out using 5% Blocking buffer (5% Blotting-Grade Blocker in 1 × PBS-T) for an hour. The membrane was washed with 1 × PBS-T before incubation with primary antibodies for an hour. The primary antibodies used depends on the respective set of experiments. The membrane was probed with primary antibody against the house-keeping protein ($\upbeta$-actin) for another hour. This is followed by the one-hour incubation with secondary antibodies conjugated with the horse radish peroxidases. Washing was performed between each of the incubation step using the 1 × PBS-T washing buffer. A final wash was done and the substrate for chemiluminescence was added. The chemiluminescence emitted was then captured by the ChemiDoc MP Imaging System. Intensity of the bands were analysed by Fiji^30^.

### Confocal immunofluorescence

DLD-1 cells (0.2 million) were plated onto a 12 mm coverslip into each well of a 24-well plate. Cells were transfected separately with the plasmid containing the WT NPR-A or the receptor isoforms. For negative control, cells were transfected with empty plasmid pCMV4. Subsequently, the transfected cells were incubated with 20 $\upmu$ l of primary antibody (Ab 14,356) coupled with 5% BSA and 1 × PBS for 1 h. Ab14356 targets the sequence LKQLKHLAYEQFNFT in the extracellular domain of NPR-A and it is able to detect all three receptor isoforms. This was followed by fixation for 15 min at 37 °C using 4% PFA. Thereafter, the cells were co-stained with 20 $\upmu$ l of diluted secondary antibody (5% BSA/1 × PBS) and wheat germ agglutinin (WGA) for another hour. The secondary antibody used was a goat anti-rabbit polyclonal antibody that was conjugated with Alexa Fluor® 488 (AF488) (ab150077). This stained the NPR-A/isoforms green while the WGA stained the glycoproteins on the cell surface red. Hence, any co-localisation of NPR-A/isoforms and WGA is indicated by yellow. In between each incubation step, the cells were washed with cold 1 × PBS for three times. Subsequently, the cells were permeabilised at 0.2% Triton X-100 for 30 min at room temperature before staining with 300 $\upmu$ l of DAPI. DAPI stained the nucleus blue. Finally, the coverslips were mounted onto a glass slide using the ProLong™ Gold Antifade Mountant reagent. The fluorescence emitted were measured using the confocal microscope. Both section images and stacked images were taken. Each fluorescence (green, blue and red) was recorded separately, and a merged image/video was also recorded. The average maximum intensity of each reading was quantified using the Imaris software. Colocalization of pixels in red channel (WGA) and green channel (NPR-A/isoforms) were analysed using Fiji^30^.

### Supplementary Information

Supplementary Information 1.

Supplementary Video 1.

Supplementary Video 2.

Supplementary Video 3.

Supplementary Video 4.

Supplementary Video 5.

Supplementary Video 6.

## Data Availability

The nucleotide sequences for NPR-A genes are available on NCBI GenBank under accession numbers NC_000001 REGION: 153,678,688..153693992 (human), NC_040252.1 REGION: 110,280,323..110294448 (sheep), NC_051337.1 REGION: 175,934,181..175950118 (rat), and NC_000069.7 REGION: 90,357,898..90373235 (mouse). The protein sequences for NPR-A are available on NCBI GenPept under accession numbers NP_000897.3 (human), XP_027831258.1 (sheep), NP_036745.1 (rat), and NP_032753.5 (mouse).

## References

[1] Wilkins MR, Nunez DJ, Wharton J (1993). The natriuretic peptide family: turning hormones into drugs. J. Endocrinol..

[2] Kuhn M (2003). Structure, regulation, and function of mammalian membrane guanylyl cyclase receptors, with a focus on guanylyl cyclase-A. Circ. Res..

[3] Potter LR, Abbey-Hosch S, Dickey DM (2006). Natriuretic peptides, their receptors, and cyclic guanosine monophosphate-dependent signaling functions. Endocr. Rev..

[4] Suga S (1992). Receptor selectivity of natriuretic peptide family, atrial natriuretic peptide, brain natriuretic peptide, and C-type natriuretic peptide. Endocrinology.

[5] Fuller F (1988). Atrial natriuretic peptide clearance receptor. Complete sequence and functional expression of cDNA clones. J. Biol. Chem..

[6] Sridharan S, Kini RM (2015). Tail wags the dog: activity of krait natriuretic peptide is determined by its C-terminal tail in a natriuretic peptide receptor-independent manner. Biochem. J..

[7] Ang, W.F., Koh, C.Y. & Kini, R.M. From Snake Venoms to Therapeutics: A Focus on Natriuretic Peptides. *Pharmaceuticals (Basel)* **15** (2022). 10.3390/ph15091153 PMC9502559 36145374

[8] Sridharan S, Kini RM (2018). Decoding the molecular switches of natriuretic peptides which differentiate its vascular and renal functions. Biochem. J..

[9] Rademaker MT, Scott NJA, Koh CY, Kini RM, Richards AM (2021). Natriuretic peptide analogues with distinct vasodilatory or renal activity: integrated effects in health and experimental heart failure. Cardiovasc. Res..

[10] Takahashi Y, Nakayama T, Soma M, Izumi Y, Kanmatsuse K (1998). Organization of the human natriuretic peptide receptor. A gene. Biochem. Biophys. Res. Commun..

[11] Potter LR, Hunter T (2001). Guanylyl cyclase-linked natriuretic peptide receptors: structure and regulation. J. Biol. Chem..

[12] Ohyama Y (1992). Cloning and characterization of two forms of C-type natriuretic peptide receptor in rat brain. Biochem. Biophys. Res. Commun..

[13] Francoeur F, Gossard F, Hamet P, Tremblay J (1995). Alternative splicing of natriuretic peptide A and B receptor transcripts in the rat brain. Clin. Exp. Pharmacol. Physiol. Suppl..

[14] Schroter J (2010). Homologous desensitization of guanylyl cyclase A, the receptor for atrial natriuretic peptide, is associated with a complex phosphorylation pattern. FEBS J.

[15] Tremblay J, Huot C, Koch C, Potier M (1991). Characterization of the functional domains of the natriuretic peptide receptor/guanylate cyclase by radiation inactivation. J. Biol. Chem..

[16] Chinkers M, Garbers DL (1989). The protein kinase domain of the ANP receptor is required for signaling. Science.

[17] Chinkers M, Singh S, Garbers DL (1991). Adenine nucleotides are required for activation of rat atrial natriuretic peptide receptor/guanylyl cyclase expressed in a baculovirus system. J. Biol. Chem..

[18] Tallerico-Melnyk T, Watt VM, Yip CC (1995). A novel guanylyl cyclase-A isoform: rat GC-A1 identification and mRNA localization to renal papilla and adrenal. Biochem. Biophys. Res. Commun..

[19] Irimia M (2008). Origin of introns by 'intronization' of exonic sequences. Trends Genet..

[20] Hartmann M (2008). Alternative splicing of the guanylyl cyclase-A receptor modulates atrial natriuretic peptide signaling. J. Biol. Chem..

[21] Potter LR, Hunter T (1998). Phosphorylation of the kinase homology domain is essential for activation of the A-type natriuretic peptide receptor. Mol. Cell. Biol..

[22] Krieger F, Moglich A, Kiefhaber T (2005). Effect of proline and glycine residues on dynamics and barriers of loop formation in polypeptide chains. J. Am. Chem. Soc..

[23] Kini RM, Evans HJ (1995). A hypothetical structural role for proline residues in the flanking segments of protein-protein interaction sites. Biochem. Biophys. Res. Commun..

[24] Yamamoto Y, Taniyama Y, Kikuchi M (1989). Important role of the proline residue in the signal sequence that directs the secretion of human lysozyme in Saccharomyces cerevisiae. Biochemistry.

[25] Park J (2018). Single-cell transcriptomics of the mouse kidney reveals potential cellular targets of kidney disease. Science.

[26] Heidenreich PA (2022). 2022 AHA/ACC/HFSA guideline for the management of heart failure: executive summary: A report of the American college of Cardiology/American heart association joint committee on clinical practice guidelines. Circulation.

[27] Ichiki T, Dzhoyashvili N, Burnett JC (2019). Natriuretic peptide based therapeutics for heart failure: Cenderitide: A novel first-in-class designer natriuretic peptide. Int. J. Cardiol..

[28] Rademaker MT (2021). Acute decompensated heart failure and the kidney: physiological, histological and transcriptomic responses to development and recovery. J. Am. Heart Assoc..

[29] Ogawa H, Qiu Y, Ogata CM, Misono KS (2004). Crystal structure of hormone-bound atrial natriuretic peptide receptor extracellular domain: rotation mechanism for transmembrane signal transduction. J. Biol. Chem..

[30] Schindelin J (2012). Fiji: an open-source platform for biological-image analysis. Nat. Methods.

