# Supplementary material for: Unveiling the potential role of natriuretic peptide receptor a isoforms in fine-tuning the cGMP production and tissue-specific function
Source: Sci Rep. 2023 Nov 22;13:20439. doi: 10.1038/s41598-023-47710-8 (PMC10665444; doi:10.1038/s41598-023-47710-8)
Supplement: Supplementary file 1 — Supplementary Information 1. [file 41598_2023_47710_MOESM1_ESM.pdf]

# Unveiling the Potential Role of Natriuretic Peptide Receptor A Isoforms in Fine-tuning the cGMP Production and Tissue-Specific Function

**Wei Fong Ang[1,2], Dan Liao[3], Cho Yeow Koh[3,*] and R. Manjunatha Kini[1,2,4,*]**

[1]Department of Biological Sciences, Faculty of Science, National University of Singapore, Singapore 117558

[2]NUS Graduate School of Integrative Sciences and Engineering, National University of Singapore, Singapore 119077

[3]Department of Medicine, Yong Loo Lin School of Medicine, National University of Singapore, Singapore 117559

[4]Department of Pharmacology, Yong Loo Lin School of Medicine, National University of Singapore, Singapore 117600

*Co-corresponding Authors:

C.Y.K. email: choyeow@nus.edu.sg

R.M.K. email: dbskinim@nus..edu.sg

**List of Supplementary Videos**

**Supplementary Video 1:** 3D z-stack of confocal microscopy images of transfected cells stained with WGA (red) and DAPI (blue).

**Supplementary Video 2.** 3D z-stack of confocal microscopy images of untransfected cells stained with WGA (red) and DAPI (blue).

**Supplementary Video 3.** 3D z-stack of confocal microscopy images of WT NPR-A transfected cells stained with WGA (red), antibody against NPR-A (green), and DAPI (blue).

**Supplementary Video 4.** 3D z-stack of confocal microscopy images of Isoform 1 transfected cells stained with WGA (red), antibody against NPR-A (green), and DAPI (blue).

**Supplementary Video 5.** 3D z-stack of confocal microscopy images of Isoform 2 transfected cells stained with WGA (red), antibody against NPR-A (green), and DAPI (blue).

**Supplementary Video 6.** 3D z-stack of confocal microscopy images of Isoform 3 transfected cells stained with WGA (red), antibody against NPR-A (green), and DAPI (blue).

## Supplementary Figures

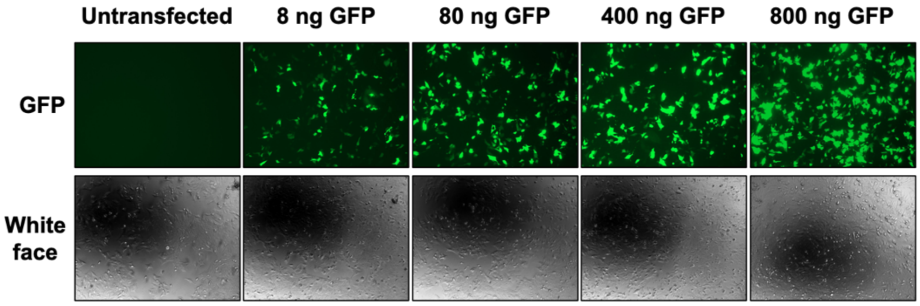


**Supplementary Figure S1.** GFP transfection efficiency test. Top panel: increasing concentration of GFP plasmid was transfected into the DLD-1 cells and the fluorescence emitted was captured using a fluorescence microscopy. Bottom panel: image of cells under white light.

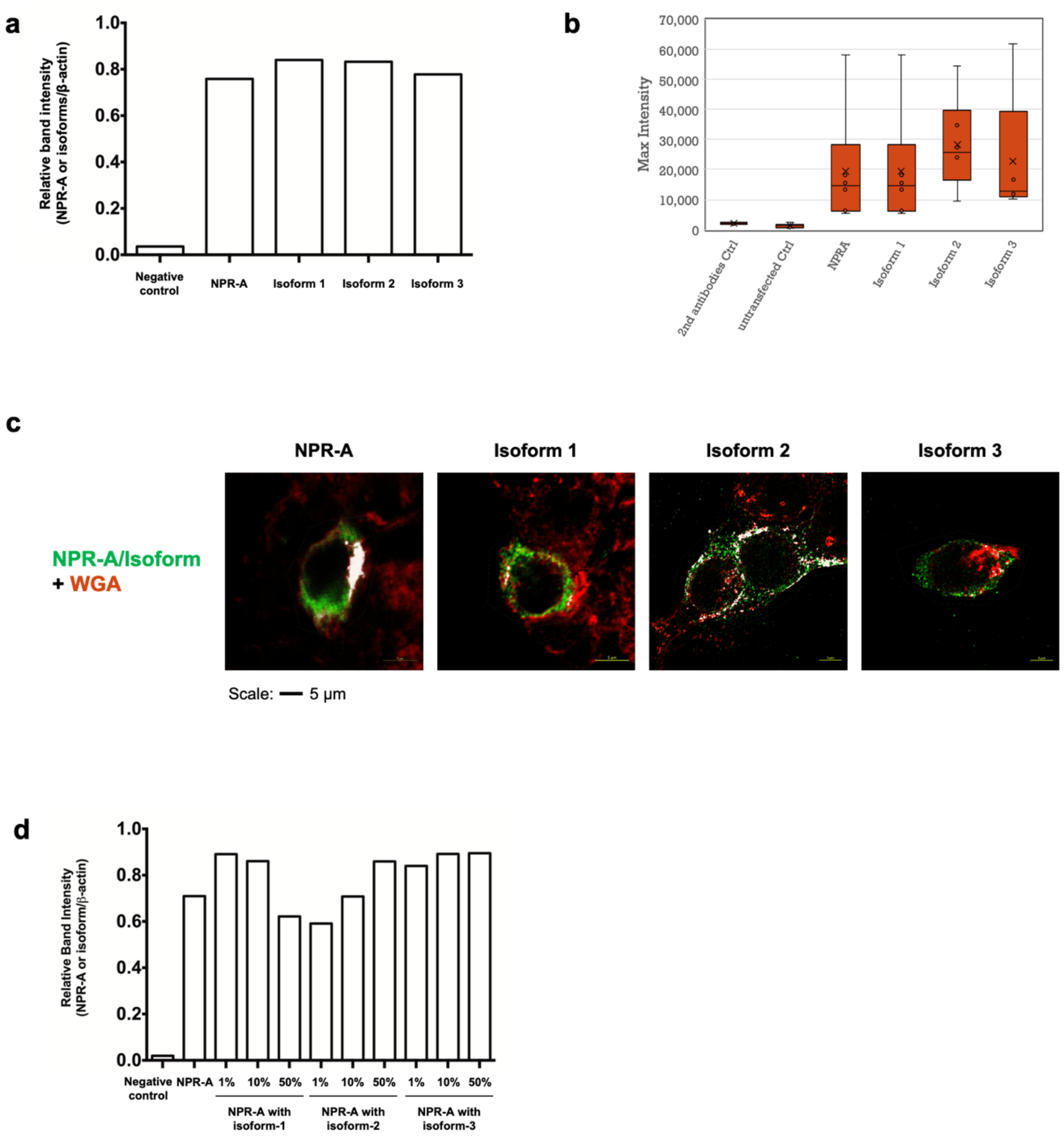


**Supplementary Figure S2.** WT NPR-A and receptor isoform 1, 2 and 3 were expressed on the cell surface. **(a)** Quantification of bands from Western blot shown in Figure 3A demonstrating similar level of expression for NPR-A and isoforms. **(b)** Maximum intensity of the fluorescence emitted from NPR-A staining were quantified using Imaris software; N=6. **(c)** Immunofluorescence staining of cells transfected with WT NPR-A or the respective receptor isoforms. Co-localization analyses were performed using Fiji ImageJ. Red: WGA, green: NPR-A/isoform, white: pixels with high intensity for both red and green signals. Scale: 5 µm. **(d)** Quantification of bands from Western blot shown in Figure 4A demonstrating similar level of expression for NPR-A and isoforms.

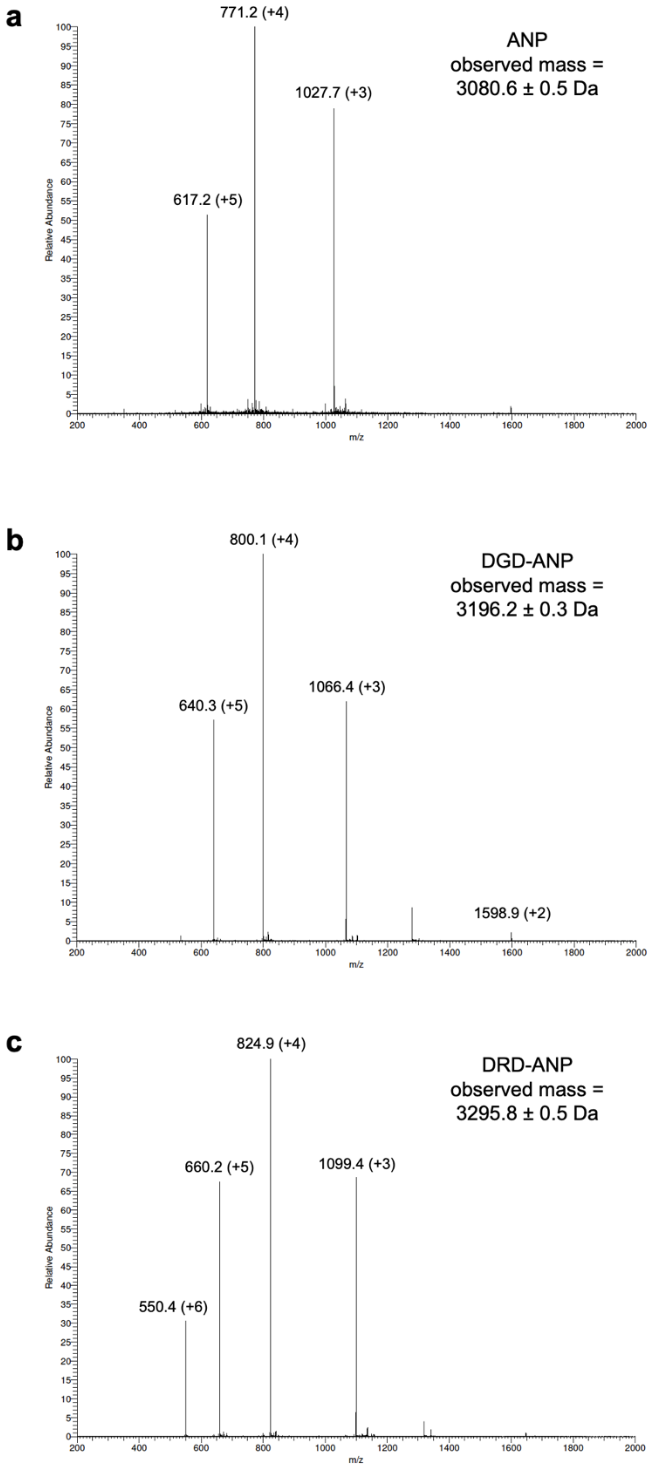


**Supplementary Figure S3.** Electrospray ionization mass spectra of synthetic peptides and their observed masses. **(a)** ANP. **(b)** DGD-ANP. **(c)** DRD-ANP.

a b

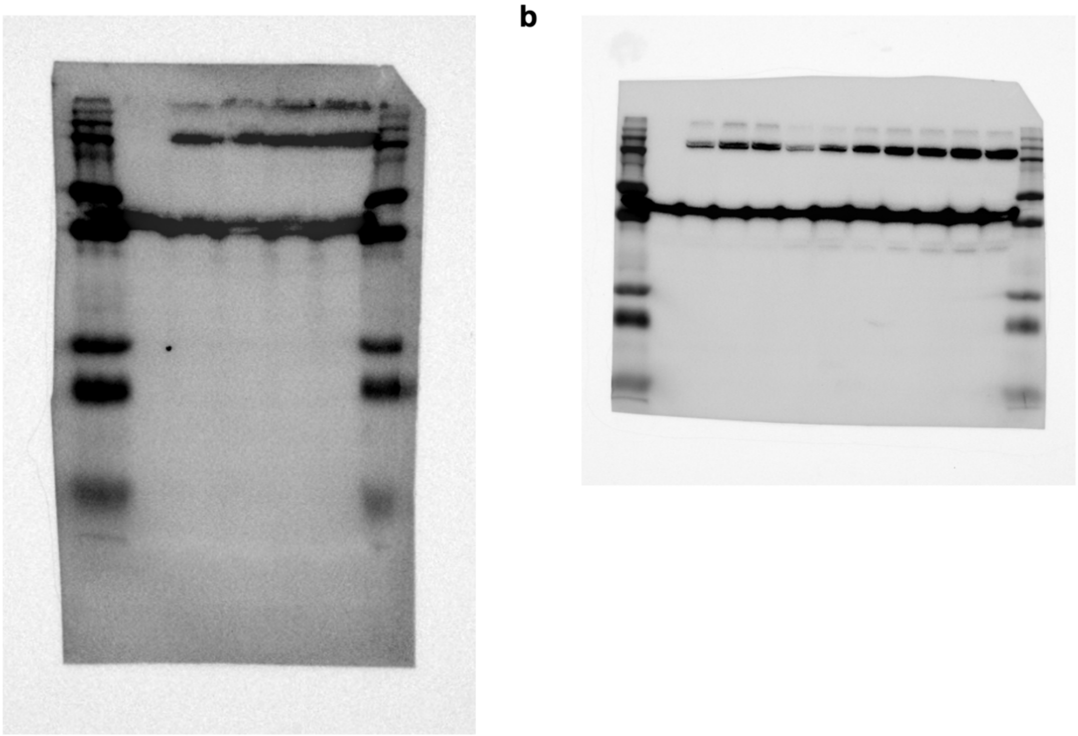

**Supplementary Figure S4.** Full-size blots for Western blot presented in the manuscript. **(a)** Correspond to Fig. 3a. **(b)** Correspond to Fig. 4a.
